# Ipilimumab, -omics, and head and neck cancers—update in 2025

**DOI:** 10.3389/fimmu.2026.1737862

**Published:** 2026-02-27

**Authors:** Robert Kucharski, Adam Kosiński, Leszek Kalinowski, Karolina Kaźmierczak-Siedlecka

**Affiliations:** 1 Department of Medical Laboratory Diagnostics – Fahrenheit Biobank BBMRI.pl, Medical University of Gdansk, Gdańsk, Poland; 2 Neodentica Dentistry Center, Gdansk, Poland; 3 Division of Clinical Anatomy, Medical University of Gdansk, Gdańsk, Poland; 4 Academy of Applied Medical and Social Sciences, Elblag, Poland; 5 BioTechMed Center, Department of Mechanics of Materials and Structures, Gdansk University of Technology, Gdansk, Poland

**Keywords:** head and neck cancers, immune checkpoint inhibitors, ipilimumab, microbial metabolites, microbiome, nivolumab

## Abstract

Immunotherapy employing immune checkpoint inhibitors (ICIs) represents a pivotal approach for the management of recurrent and metastatic head and neck cancers (HNCs). Ipilimumab is a fully human monoclonal IgG1κ antibody against cytotoxic T-lymphocyte antigen-4 (CTLA-4), which can be introduced as a monotherapy or dual immunological regimen with nivolumab (anti-programmed death protein 1, PD-1). The background of the use of these monoclonal antibodies as combination immunotherapy is strongly associated with their different mechanisms of action. CTLA-4 and PD-1 are able to regulate the function of T cells through different mechanisms. Despite the better efficacy of immunotherapy with ipilimumab + nivolumab in HNCs observed in some cases, the overall effect regarding the comparison of ipilimumab *versus* ipilimumab + nivolumab is still unclear. The microbiome is one of the biomarkers that affect the response to immunotherapy with ICIs, including ipilimumab. Nevertheless, there is a clear lack of data in this context with regard to HNCs. The beneficial signature of the microbiome contributes to the prevention of the immune-related adverse events caused by ipilimumab. Notably, the incidence of gastrointestinal side effects induced by ICIs is significantly increased in the dual regimen with ipilimumab + nivolumab, which affects its recommendation for patients with HNCs

## Graphical Abstract

**Figure:**
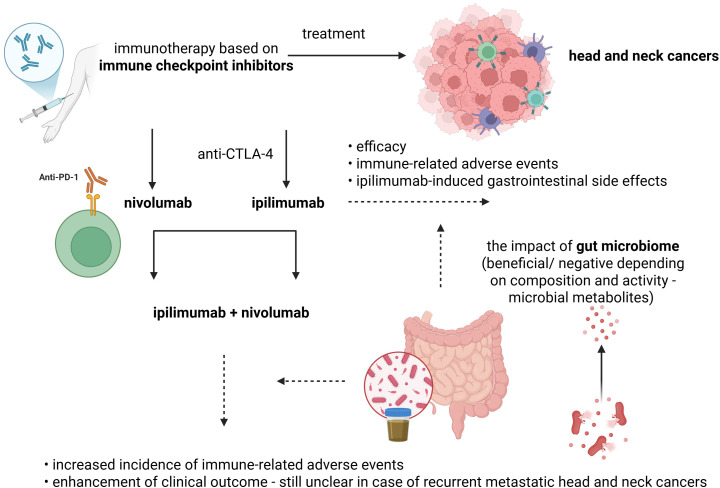

## Highlights

- Ipilimumab, which targets CTLA-4, is utilized for recurrent/metastatic head and neck cancers (HNCs) as a monotherapy or combined with nivolumab. CTLA-4 inhibitors demonstrate comparatively lower activity in HNCs, potentially due to tumor hypoxia impairing T-cell infiltration.
- Combination therapy (anti-CTLA-4 + anti-PD-1) offers a broader immunological approach; however, superior clinical outcomes over monotherapy remain uncertain, and the heightened immune-related adverse events (irAEs) necessitate careful patient selection.
- The microbiome may modulate both the efficacy and the toxicity of ipilimumab, particularly in preventing gastrointestinal irAEs; however, HNC-specific data are limited.

## 1 Introduction

The category of biologic drugs comprises monoclonal antibodies, recombinant proteins, enzymes, and cytokines. Multiple formats of both antibodies and antibody derivatives are available, for instance, full-length antibodies or antibody fragments only, such as Fab, Fc, and scFv (single-chain variable fragments), and other formats [e.g., antibody–drug conjugates (ADCs), chimeric antigen receptor (CAR) T cells, and bispecific T-cell engagers) (1, 2). There are some crucial differences between biologic drugs and small-molecule medications in terms of, among others, molecular weight (biologics: even more than 1,000 kDa; small-molecule drugs: <1 kDa). Overall, biologic drugs are not appropriate for oral administration. This is due to their physical instability, digestion, and deactivation prior to their absorption into the blood stream. Therefore, two other methods are recommended: 1) intravenous infusion (100% bioavailability; for acute conditions, a high *C*_max_ is recommended for therapeutic effect) and 2) subcutaneous injection (<100% bioavailability; generally recommended for chronic conditions) (1). The inability to deliver monoclonal antibodies to an appropriate amount of both cancer cells and tumor-infiltrating cells means that intratumoral injections with monoclonal antibodies still being tested (3). Intratumoral delivery, although not Food and Drug Administration (FDA)-approved, is under investigation for use with low-dose drugs and to enhance local immunostimulation while reducing systemic toxicity (3–5).

**Figure 1:**
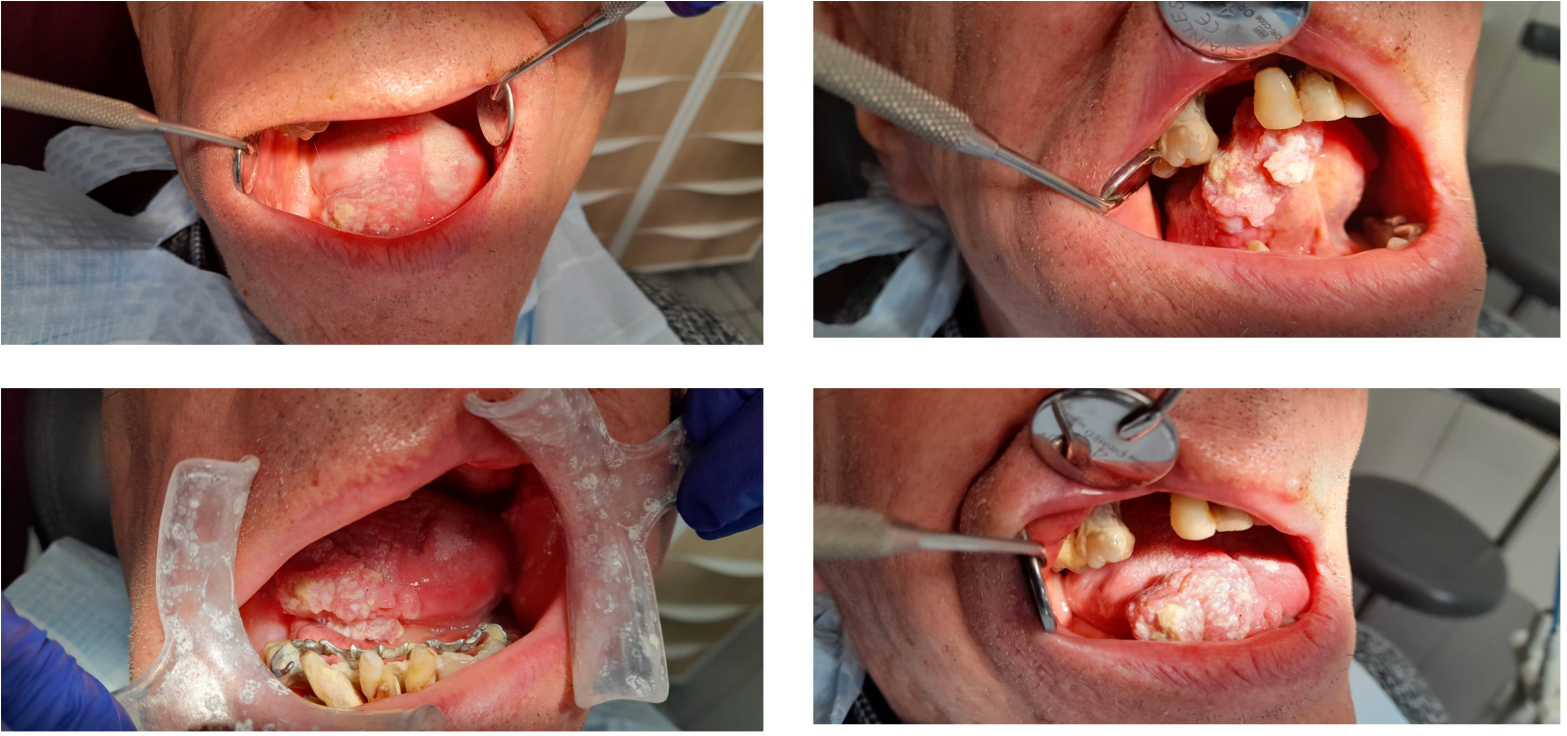
Case of squamous cell carcinoma of the tongue with infiltration into the floor of the mouth. Head and neck cancers (HNCs) are often diagnosed at an advanced stage with metastatic sites. The assessment is based on the TNM tool (T, tumor; N, nodes; and M, metastasis). Photographs were taken from a dental practice, Neodentica Dentistry Center, Gdansk, Poland, and provided by the attending oral surgeon. This figure was created using Biorender.com.

Monoclonal antibodies possess complex pharmacokinetic and pharmacodynamic profiles relative to conventional chemotherapeutics (6). Their integration into clinical oncology has reshaped therapeutic strategies, including for head and neck cancers (HNCs), a heterogeneous group of neoplasms arising from the oral cavity, pharynx, larynx, sinonasal cavities, lips, and salivary glands (7, 8). Squamous cell carcinoma accounts for ~90% of cases (**Figure 1**). HNCs represent the sixth most commonly diagnosed cancers worldwide, often presenting at advanced stages with infiltration of local spaces (e.g., pterygomandibular, submandibular, and sublingual) and high mortality rates, underscoring the urgent need for novel treatments, including immunotherapy with ipilimumab (9). This mini-review focuses on: i) monotherapy with ipilimumab *versus* combination immune checkpoint inhibitor (ICI) regimens and ii) the influence of -omics, particularly the microbiome, on therapeutic outcomes.

## 2 Ipilimumab: basic characteristics and mechanism

Ipilimumab, developed at the University of California, Berkeley, is a fully human IgG1κ monoclonal antibody targeting CTLA-4 (cytotoxic T-lymphocyte antigen-4) and CD152 (cluster of differentiation 152) (10, 11). CTLA-4 downregulates the T-cell responses and induces tolerance, primarily via interactions with CD80/CD86 on antigen-presenting cells (10). The background of immunotherapy includes an appropriate pair of ICIs (on T cells) and receptors (on tumor cells), for instance, CTLA-4 combined with CD86, CD80, B7-1, or B7-2 and programmed death protein 1 (PD-1) inhibitor combined with programmed death-ligand 1 (PD-L1), PD-L2, B7-H1, or B7-H212. Some of the immunosuppressive activities of CTLA-4 depending on cell type are as follows: i) regulatory T cells (Tregs)—decreasing the presentation of antigen and the activation of T cells as a result of interaction with CD80 and CD86 on antigen-presenting cells; ii) Tregs—co-expression of CD39 and CD73 resulting in the reduction of T cells activating adenosine 5′-triphosphate; and iii) mature myeloid dendritic cells—activity observed in the tumor microenvironment as the release of vesicle-packaged CTLA-4 (12). The complementary engagement of ICIs with tumor and immune cell receptors—e.g., CTLA-4 with CD80/CD86 and PD-1 with PD-L1/PD-L2—forms the mechanistic basis for immunotherapeutic strategies.

## 3 Combination ICIs (ipilimumab + nivolumab) *versus* monotherapy with ipilimumab

ICIs are used as components of therapeutic strategies for the treatment of recurrent metastatic head and neck squamous cell carcinoma (HNSCC) (13, 14). As mentioned above, there is still an open question regarding which one is more effective: combination immunotherapy with ICIs or monotherapy. The justification for the use of the combination of anti-CTLA-4 and anti-PD-1 is based on their different mechanisms of action. T-cell function is regulated by CTLA-4 and PD-1 through different methods, as follows: 1) anti-PD-1 mainly causes both the expansion and the recruitment of existing antitumor T cells, whereas anti-CTLA-4 acts through the generation of new T-cell clones; and 2) anti-PD-1 does not induce the subset of Th1-like CD4^+^, whereas anti-CTLA-4 is able to do this (15). Vos et al. (14) investigated the efficacy of neoadjuvant monotherapy with nivolumab [first-in-class fully human immunoglobulin G4 (IgG4) and anti-PD-1]/ipilimumab + nivolumab prior to surgery in patients with HNSCC (*n* = 32; histologically confirmed T2–T4, N0–N3b, M0 primary/recurrent, resectable HNSCC) in a non-randomized phase Ib/IIa trial. A major pathological response (MPR) was noted in 35% of patients treated with combination ICIs (the rate in monotherapy with nivolumab was 17%). In addition, the 24-month median postsurgical follow-up revealed that none of the MPR patients developed recurrent HNSCC. Therefore, the combination therapy of ipilimumab + nivolumab appears a promising option for these patients (14). Evaluation of the data presented during the American Society of Clinical Oncology (ASCO) and the European Society of Medical Oncology (ESMO) meetings in 2021 revealed that overall survival was not improved after treatment with the combination of nivolumab + ipilimumab (as palliative first-line therapy) (16). Harrington et al. (17) presented the results of a phase 2 CheckMate 714 randomized clinical trial on patients with recurrent or metastatic HNSCC. Participants were divided to receive nivolumab + ipilimumab (3 mg/kg intravenously every 2 weeks and 1 mg/kg intravenously every 6 weeks, respectively) or nivolumab (with the same dose) + placebo for up to 2 years or until disease progression (other reasons: toxic effects that were not acceptable or consent withdrawal). An acceptable safety profile was observed for nivolumab + ipilimumab (17). Despite some benefits of this type of combination immunotherapy, the general efficacy in the case of metastatic HNSCC is still unclear. Similar observations were noted for advanced salivary gland cancers. Vos et al. (18) conducted a phase 2 trial including 64 patients with metastatic salivary gland cancers (SGCs) [classified according to histological assessment: adenoid cystic carcinoma (cohort 1) and other types of SGCs (cohort 2)]. Participants were treated with the combination immunotherapy of nivolumab + ipilimumab (intravenously, 3 mg kg^−1^ every 2 weeks and 1 mg kg^−1^ every 6 weeks, respectively). The following were the main endpoints: efficacy ≥4 objective responses: 6% in cohort 1 and 16% in cohort 2; median progression-free survival: 4.4 months in cohort 1 and 2.2 months in cohort 2. In cohort 1, the efficacy of this type of immunotherapy was limited, whereas it can be a promising option for other SGCs (especially for patients with salivary duct carcinomas) (18). Overall, while dual-ICI therapy may offer immunological advantages, the clinical benefits vary by tumor type and remain incompletely defined. Besides ipilimumab and nivolumab, the use of pembrolizumab (a PD-1 inhibitor) is significant in the treatment of HNSCC (especially as a neoadjuvant therapy). Notably, pembrolizumab is recommended as a first-line treatment: 1) in the form of monotherapy for recurrent/metastatic HNSCC (in PD-L1-positive disease) and 2) in combination with chemotherapy (platinum + fluorouracil) without the influence of the PD-L1 status (19). Recently, in 2025, an improvement in the event-free survival of patients with locally advanced HNSCC was observed after the addition of pembrolizumab (neoadjuvant/adjuvant) to standard care (20).

## 4 -Omics and ipilimumab response

There are a number of biomarkers that are able to affect the response to immunotherapy based on ICIs, such as the expression of PD-L1, the number of mutations in the cancer cell DNA (mutations per megabase, also known as the tumor mutation burden), the microsatellite instability, interferon gamma, and circulating exosomes (11, 21). Elimination of the primary tumor response to immunotherapy can be caused by surgical ablation/broad radiation of tumor-draining lymph nodes (22). The ablation of lymph nodes inhibits the activity of anti-PD-1 and anti-CTLA-4 to control tumor growth. Both the gut and tumor microbiomes also affect the efficacy of immunotherapy based on ICIs (23). Orally administered live *Akkermansia muciniphila* is the most effective form for the activation of CD8 T cells (in a mouse cancer model) compared with pasteurized *A. muciniphila* and the *A. muciniphila* membrane protein Amuc_1100 (24–27). It should be emphasized that, based on anti-PD-1/PD-L1, *A. muciniphila* improves immunotherapy (25). The data on the effects of the microbiome-related aspects on the efficacy of ipilimumab are strongly limited, particularly in HNCs. It has been reported that distinct *Bacteroides* species influence the antitumor effects of CTLA-4 blockade (28). Recently, it has been shown that immunotherapy based on the dual regimen of nivolumab + ipilimumab can be enhanced by live *Clostridium butyricum* in the case of metastatic renal cell carcinoma (29). Management with a regimen combining nivolumab (3 mg/kg i.v. every 3 weeks through 12 weeks) + ipilimumab (1 mg/kg i.v. every 3 weeks through 12 weeks) + bifidogenic live bacterial product (i.e., CBM588, at 80 mg orally twice a day) is associated with a significantly longer progression-free survival compared with an immunological regimen without microbe administration (12.7 *versus* 2.5 months, *p* = 0.001) (30). Nevertheless, this effect was observed in patients with metastatic renal cell carcinoma, similarly to the case mentioned above (30). Potential enhancement of immunotherapy based on ICIs can also be obtained with microbial metabolites, e.g., desaminotyrosine, which is able to improve the efficacy of anti-CTLA-4 through the IFN-I signaling pathway (31).

The most common gastrointestinal complications induced by ipilimumab + nivolumab include diarrhea, nausea, a reduced appetite, vomiting, constipation, colitis, and abdominal pain (32). Ipilimumab induced diarrhea in 33% of patients and colitis in 7% of cases (33). These results are similar when taking into consideration the dual regimen including nivolumab + ipilimumab (diarrhea: 21%–37% of patients, colitis: 4%–8%) (33). However, a systematic review and meta-analysis revealed that ipilimumab + nivolumab is associated with a higher risk of colitis [any grade, relative risk (RR) = 4.52] and diarrhea (RR = 1.68) compared with monotherapy with nivolumab (34). With regard to the microbiome, it has been observed that colitis-resistant patients present greater abundance of Bacteroidetes, suggesting the protective role of these microbes (35). Notably, some microorganisms, such as Lachnospiraceae spp. and *Streptococcus* spp., can be implicated in the development of irAEs (in the case of ipilimumab) (36). Therefore, the effect of the microbiome in the context of ipilimumab-induced adverse events may be beneficial or pathogenic dependent on the microbiome signature.

It should also be emphasized that the term -omics covers different types of “-omics,” such as transcriptomics, proteomics, and metagenomics, among others. As above, microbiome-related aspects have been discussed in the context of response to immunotherapy based on ICIs. In 2025, a comprehensive analysis of 16S rRNA amplicon and metagenomic sequencing data was performed to provide a blueprint of the oral microbiome in patients with HNSCC (37). Oral swabs were taken from patients with different types of pathological changes: benign lesions (*n* = 56), precancerous lesions (*n* = 29), early-stage HNSCC (*n* = 39), and late-stage HNSCC (*n* = 48). The construction of an HNSCC diagnostic classifier was prepared using machine learning-based tools. A dysbiotic microbial–metabolic signature indicates a potential risk factor for the progression of HNSCC (37). Notably, a systematic review (38) prepared according to the PRISMA (Preferred Reporting Items for Systematic Review and Meta-Analyses) statement guidelines provided data on the type of microbial dysbiosis in HNSCC. At the genus level, microbes such as *Fusobacterium*, *Peptostreptococcus*, *Alloprevotella*, *Capnocytophaga*, *Catonella*, and *Prevotella* were differentially increased in HNSCC, whereas others were reduced (i.e., *Rothia*, *Actinomyces*, *Veillonella*, and *Streptococcus*). In addition, HNSCC is positively correlated with periodontal pathogens but negatively associated with commensal bacteria. Differential enrichment of the pro-inflammatory genomic pathways was analyzed using metagenomic screening of the microbiome. The specific signature of the oral microbiome can be included into both the screening and the diagnosis of HNSCC (38). Due to the fact that the decreased levels of some microbes in the saliva (*Eubacterium infirmum*, *Actinobaculum*, and *Selenomas*) can be related to non-response to neoadjuvant immunotherapy in patients with oral squamous cell carcinoma, salivary metagenome sequencing can also be useful in the analysis of the response to immunotherapy (39).

## 5 Discussion and conclusions

ICIs, including ipilimumab, offer promising avenues for the management of HNCs. Combination therapy with nivolumab may provide synergistic immunological benefits, particularly in PD-L1-negative tumors, but also increases irAE incidence. Current evidence does not definitively favor combination therapy over monotherapy, highlighting the need for patient-specific risk–benefit assessments. Besides nivolumab, pembrolizumab (as a monotherapy or in combination with chemotherapy) is also recommended for the treatment of HNCs. It can improve survival (especially in the case of locally advanced HNSCC). Despite the beneficial effects of both nivolumab and pembrolizumab, the treatment of HNC is still a challenge, and better treatment approaches need to be investigated. -Omics insights, particularly the microbiome composition, may modulate both efficacy and toxicity; however, HNC-focused data remain sparse. Future investigations should integrate multi-omic analyses and microbiome-directed interventions in order to optimize ICI therapy for HNCs, balancing therapeutic gain against potential immune-mediated toxicities.
